# Differences between willingness to pay and willingness to accept for visits by a family physician: A contingent valuation study

**DOI:** 10.1186/1471-2458-10-236

**Published:** 2010-05-10

**Authors:** Jesús Martín-Fernández, Ma Isabel del Cura-González, Tomás Gómez-Gascón, Juan Oliva-Moreno, Julia Domínguez-Bidagor, Milagros Beamud-Lagos, Francisco Javier Pérez-Rivas

**Affiliations:** 1 San Martín de Valdeiglesias Health Center. 8th Primary Care Area Madrid Health Service. Spain; 2 Research Unit, 9th Primary Care Area, Madrid Health Service, Madrid, Spain; 3 Rey Juan Carlos I University, Madrid, Spain; 4 Puerta Bonita II Health Center. 11th Primary Care Area. Madrid Health Service, Madrid, Spain; 5 Complutense University of Madrid, Madrid, Spain; 6 Economic Analysis Department, Castilla la Mancha University, Spain; 7 CIBERESP-CIBER In Epidemiology and Public Health, Spain; 8 Quality Unit. 11th Primary Care Area, Madrid Health Service, Spain; 9 Research Unit, 11th Primary Care Area, Madrid Health Service, Spain

## Abstract

**Background:**

The economic value attributed by users of health services in public health systems can be useful in planning and evaluation. This value can differ from the perspectives of Willingness to Pay (WTP) and Willingness to Accept [Compensation] (WTA).

Our objective was to study the perceptions of the patient about the service provided by the family physician by means of the WTA/WTP ratio.

**Methods:**

An economic evaluation study by the Contingent Valuation Method was designed. Interviews were conducted with 451 subjects at six health centres (four urban and two rural) in areas with different socioeconomic characteristics. A payment card was used to measure the WTP and WTA. Other characteristics of the subject or service that could influence these responses were collected. An explicative model was constructed to study the WTA/WTP relationship.

**Results:**

Four hundred and four subjects (89.6%) expressed a WTP and WTA different from zero. The WTA/WTP quotient showed a median of 1.55 (interquartile range 1-3.08) and a mean of 3.30 (IC 95%: 2.84-3.75). The WTA/WTP ratio increases with age and in low-income areas. It decreases in professional groups with more specialized activities, with growing family income, and in the chronically ill. Other characteristics related to the perception of state of health, accessibility to the service, satisfaction, or perception of risk were not explicative.

**Conclusions:**

Subjects who were older and had a less favourable socioeconomic situation expressed a higher WTA/WTP ratio when valuing the visit to the family physician. These characteristics could identify a profile of "aversion to loss" with respect to this service.

## Background

The calculation of the value of health care, and its implications for the distribution of economic resources, is an element of constant debate [1]. To place a value on the service the user receives in the health system is complicated, there being no market that enables this. There are methodologies, such as Contingent Valuation (CV), developed in the framework of cost-benefit analysis, which are essential to attribute value to goods or services that cannot be exchanged in a market [2].

The value attributed by CV methodology to a good or service can be studied from the perspective of willingness to pay (WTP), the maximum amount a person would be willing to offer for a good, or by the willingness to accept compensation (WTA), the minimum monetary amount required for an individual to forgo some good, or to bear some harm. The relationship between the sum of WTP in subjects affected by an actuation or change in a service, and WTA of those affected by the same change, constitutes a decision criteria in cost-benefit analysis. An actuation or change in a service can be evaluated as acceptable or beneficial, if the sum of individual's WTP for the change exceeds the sum of the WTA for the change (Kaldor-Hicks or potential Pareto criterion). This criteria assumes that WTA to avoid the loss of a good or service and WTP to have access to a good or service, expressed by the same subject, must be similar. However, we know that the values obtained by WTA are consistently higher than those expressed by WTP, when valuing the same good [3-7].

The difference between WTA and WTP for the same good or service has been widely studied through both theory and experiments [8]. Economic theory has attributed the differences found in valuing a good or service by WTA and WTP to an "income effect". The payment capacity is reached before satisfaction with the compensation is perceived. So WTA-WTP differences must be small since they vary only by an income effect. However, Hanemann proposed that the differences between WTA and WTP could be arbitrarily large (infinite in the limit), depending on the degree of substitutability between the non-market good and other ordinary market commodities [9,10]. Other authors claim that the difference between WTA and WTP expressed for the same good may be due to a hypothetical bias. So, the less information there is about the good evaluated and the higher the costs of information, greater is the bias, and greater the WTA/WTP ratio [11]. Perhaps the most studied theory turns around "loss aversion". The basic idea behind loss aversion is that losses are weighted far more heavily than gains. The point of reference for the loss and gain is an "endowment point". Valuations of gains and losses are always relative to the reference or endowment point, losses are valued more heavily than gains, and the valuation function exhibits diminishing marginal valuation the further away from the reference point one gets. Depending on the degree of loss aversion, WTA could greatly exceed WTP [12]. The explanation of this theory is essentially psychological, so that theory is essentially being advanced as an explanation of observed behaviour. Economic theory tries to explain this resistance to loss abounding in the reasons of information costs, the uncertainty about the value of the good and the impossibility of reversing the processes of loss [13]. These approaches to understanding the phenomenon, supported by experimental results, are not mutually incompatible [14].

Although contingent valuation has is situated in cost-benefit analysis, the relationship between WTA and WTP has considerable importance in other types of studies. In cost-effectiveness analysis the rules of decision are based on the acceptability of the incremental cost per unit of effectiveness. This could be interpreted to mean that this cost would be acceptable or not depending on the estimated societal willingness to pay (WTP) for an additional unit of health effect. A WTA/WTP ratio greater than one means that the utility perceived by the loss is greater than that perceived by an equivalent gain. This, in turn, has implications for the threshold at which to declare an intervention to be cost-effective, depending on whether it represents an increase in the utility with a cost increase (first quadrant of the cost-effectiveness chart) or a loss of utility with lower costs (third quadrant of the cost-effectiveness chart) [14]. An effort has been made to resolve this problem estimating what the "kink" in threshold of acceptability must be depending on whether we are valuating losses or benefits in the field of health [15,16], but this issue has not been fully resolved.

The valuation of certain health services from the perspective of WTA and WTP can be useful in health planning. The differences between WTA and WTP can help to understand not only the value attributed to a service but also the capacity to substitute it or the resistance to its loss.

Health care in our setting is organized in a national health system. The providing of services takes place at two traditionally differentiated care levels: primary care (PC) and specialized care. The entire system is financed fundamentally by taxes, with no direct cost at time of use. This is why the manifestation of the needs expressed by health demand can be distorted. Health services utilization can be influenced by other factors than health needs, specially in PC [17,18]. Therefore, it will be necessary to include other elements, in addition to the need expressed by health demand, as criteria for health planning, and among these new variables we find that the perception of value the user has of the service is fundamental. We know that the user of the health system is satisfied with the care received in primary care [19] and in certain contexts the characteristics of the willingness to pay have been studied by the method of Contingent Valuation [20,21].

The purpose of this paper is to advance in the knowledge of the perceptions the user has of the visit to the family physician in a public health system, with respect to its economic value and the predisposition to the possible substitution of this service, by the analysis of the relationship between WTA and WTP.

## Methods

### Design and subjects of study

A cross-sectional study was conducted using Contingent Valuation methodology.

Subjects were randomly selected during a week from the attendance records of visits at four urban and two rural centres of the Community of Madrid, Spain. The centres were, in turn, chosen according to their location in areas in the upper terciles (3 centres) or lower terciles (3 centres) of income distribution in the Community of Madrid. An invitation to participate was extended to 487 subjects, 451 giving their consent to be surveyed. The subjects who did not accept being interviewed expressed a lack of time in twenty-seven cases, were not interested in the subject in two cases, and gave other reasons in seven cases.

The interview was conducted at each patient's own health centre, just after a visit to the family physician, outside the health care area. The fieldwork was performed by a single trained interviewer between December 2007 and March 2008. All included patients signed an informed consent and the study was approved by the Ethic review Board of the "12 de Octubre Hospital", Madrid, Spain.

### Variables studied

WTP and WTA were valued with a questionnaire devised for this purpose (see Additional file 1). The description of the scenario was done verbally, briefly explaining the purpose of the work, and two scenarios were presented. One evaluated WTP for the service just received. The second scenario asked for the WTA if this service were eliminated.

The response was made indicating on a payment card with ascending categories the group that included the person's maximum WTP or WTA. Two cards were given to determine WTP and the same two cards were subsequently used to determine WTA. The first one only contained three values: less than 20 euros; between 20 and 40 euros; more than 40 euros. The second card set limits to the value offered in the first between the categories: 0 euros; 1-5 euros; 6-10 euros; 11-15 euros; 16-20 euros; 21-25 euros; 26-30 euros; 31-40 euros; 41-50 euros; 51-60 euros; 61-70 euros; 71-80 euros; 81-90 euros; more than 90 euros.

Subsequently, the age, sex, place of birth, characteristics of health needs, accessibility to the service, the existence of other types of insurance, risk perception, relationship with the professional the person had been attended by, main activity, employment group, level of studies and family income were gathered.

To evaluate health need note was made of the existence of chronic pathologies, existence of hospital admissions, and number of visits to the family physician in the last year. The EuroQol-5D was used to approach the subjective perception of the patient's state of need.

Accessibility to the service was studied by means of the time needed to set an appointment and the waiting-time from time of appointment to visit.

To evaluate risk perception questions were asked about the existence of any of the following risky behaviours: smoking, driving vehicles without a seatbelt, risky sexual behaviour, non-observation of job-safety measures and excessive alcohol consumption.

The relationship with the family physician was evaluated with the questionnaire proposed by Van der Feltz-Cornelis and cols [22], adapted to Spanish, which produces a synthetic index from 1 (the worst state possible) to 5 (the most satisfactory relationship possible).

To evaluate the socioeconomic situation questions were asked about the highest level of studies completed (illiterate, no studies, primary, secondary, superior), for their "social class" [23] (classes I, most skilled job, to V, least skilled), and for the current employment situation (student, homemaker, retired, unemployed, active).

Average income was calculated adding together all the family unit's income weighed by family size N (Average income = family income/N^0.4^). Subjects were attributed the characteristic of residing a high or low income areas according to the centres they visited.

### Analysis

The WTP and WTA declared were transformed into a continuous variable, applying to each interval its average value for descriptive study.

The relationship between WTA and WTP was studied first in those subjects who expressed values different from zero in both scenarios.

An explicative multivariate model was constructed in which the dependent variable was the WTA/WTP ratio and the independent variables were those related to health needs, accessibility, risk perception, satisfaction with the service, socioeconomic situation and income. If there was more than one variable to measure the same characteristic the one with the higher correlation with the dependent variable was introduced first into the model, followed by those that, without showing collinearity, enhanced the explicative capacity. Given that the dependent variable (WTA/WTP) presented an asymmetrical distribution, it was smoothed applying a conversion based on its natural logarithm (ln). As an alternative, another model was constructed, in which the outcome variable for the regression analysis was the difference between WTA and WTP. The direct utilization of the difference made the construction of the model unsuitable, having a very asymmetrical distribution, and this distribution could not be smoothed by taking logarithms because one cannot operate with ln when the differences are 0 or negative numbers.

The model with the highest explicative capacity with least number of variables (parsimony principle) was selected, based on the theoretical framework explained above. A priori, the assumption of homoscedasticity was not certain. Therefore, the Eicker-White covariance matrix estimator was employed to construct the model, which provides a consistent estimator of the regression coefficients in the presence of heteroscedasticity of an unknown form [24]. Residuals analysis assured that the assumptions of the model of normality, linearity, independence and homogeneity of the variances were respected.

This analysis was repeated substituting the zero values in WTP and WTA. Thus, the "zero values" were substituted for each individual for the average WTP or WTA values observed in similar individuals. To reduce possible biases, a matching was established between each individual with zero responses and the individuals more similar, taking into account for this age, gender, level of studies, and level of income.

#### Working hypothesis

In the conceptual framework set forth, expectations were for a higher WTA/WTP relationship in those subjects with a lower socioeconomic level [8], with fewer economic resources, with less capacity to find a substitute for the good that is evaluated [9,10], with more need and less information [11], with higher satisfaction [12], and with higher uncertainty about the good evaluated [13]

## Results

Table 1 shows the characteristics of the 451 subjects who finally consented to participate in the study.

**Table 1:** Characteristics of the subjects included in the study.

	Mean (CI 95%)	Median (IQ range)	Percentages over total
**Age**	57.3 (56.0-58.7)	57.0 (45.0-70.0)	

**Sex (male/female)**			36.6%/63.4%

**Nationality (Spanish/other)**			89.6%/10.4%

**Other insurance (yes/no)**			23.5%/76.5%

**Any illness (yes/no)**			71.2%/28.8%

**VAS - EuroQol-5D**	64.4 (62.5-66.4)	50.0 (60.0-80.0)	

**N° visits to the physician/year**	15.3 (14.3-16.4)	13 (7-20)	

**Hospital admissions (yes/no)**			20.0%/80.0%

**Time in making appointment**			

Same day			26.6%

One day			33.0%

Two days			19.1%

Three days			5.1%

More than three days			16.2%

**Waiting time at visit**			

Less than 15 minutes			70.5%

Between 16 and 30 minutes			23.1%

Between 31 and 60 minutes			5.5%

More than one hour			0.9%

**Relation with family physician** **(1 worst, 5 best possible)**	4.4 (4,3-4.5)	4.8 (4,0-5,0)	

**N° of risky behaviours**	0.5 (0.4-0.6)	0.0 (0.0-1.0)	

**Education level**			

Illiterate			0.9%

No education			20.4%

Primary education			37.9%

Secondary education			25.5%

Superior education			15.3%

**Social class**			

Group I: Managers, directors			12.9%

Group II: Intermediate positions			16.0%

Group III: Skilled non-manual worker			13.5%

Group IV: Skilled (or partially skilled) manual worker			43.5%

Group V: Unskilled manual worker			14.3%

**Adjusted family income (€)**	1288.8 (1200.0-1377.6)	966.6 (682.1-1591.5)	

CI 95%: Confidence interval 95%;

IQ range: interquartíle range (percentile 25-percentile 75)

VAS-EuroQol-5D. Visual analog scale of EuroQol-5D questionnaire

Thirty-four subjects (7.5%) expressed zero WTP, seventeen (3.8%) presented zero WTA, of which four (1.0%) had also expressed a WTP equal to zero. Four hundred and four interviewees (89.6%) expressed a WTP and WTA greater than zero.

For the subjects who expressed a WTP and WTA greater than zero, the WTP presented a median of €18.0, (interquartile width €8.0-28.0, mean €21.3, CI 95%: €19.6-23.0). Median WTA was €35.5, (interquartile width €18.0-45.5, mean €37.2, CI 95%: €35.1-39.4).

The subgroup that expressed zero WTP or WTA had a mean age moderately higher than the rest (62.4 vs 56.7 years, p = 0.010), more chronic illnesses (3.3 vs 2.6 illnesses by person, p = 0.026), and a lower level of studies (17.7% illiterates or primary studies compared with 8.5% p = 0.008). There were no significant differences in grouping by gender, origin, perception of quality of life, accessibility to the service, risky behaviour, professional group, or adjusted family income.

**Figure 1:**
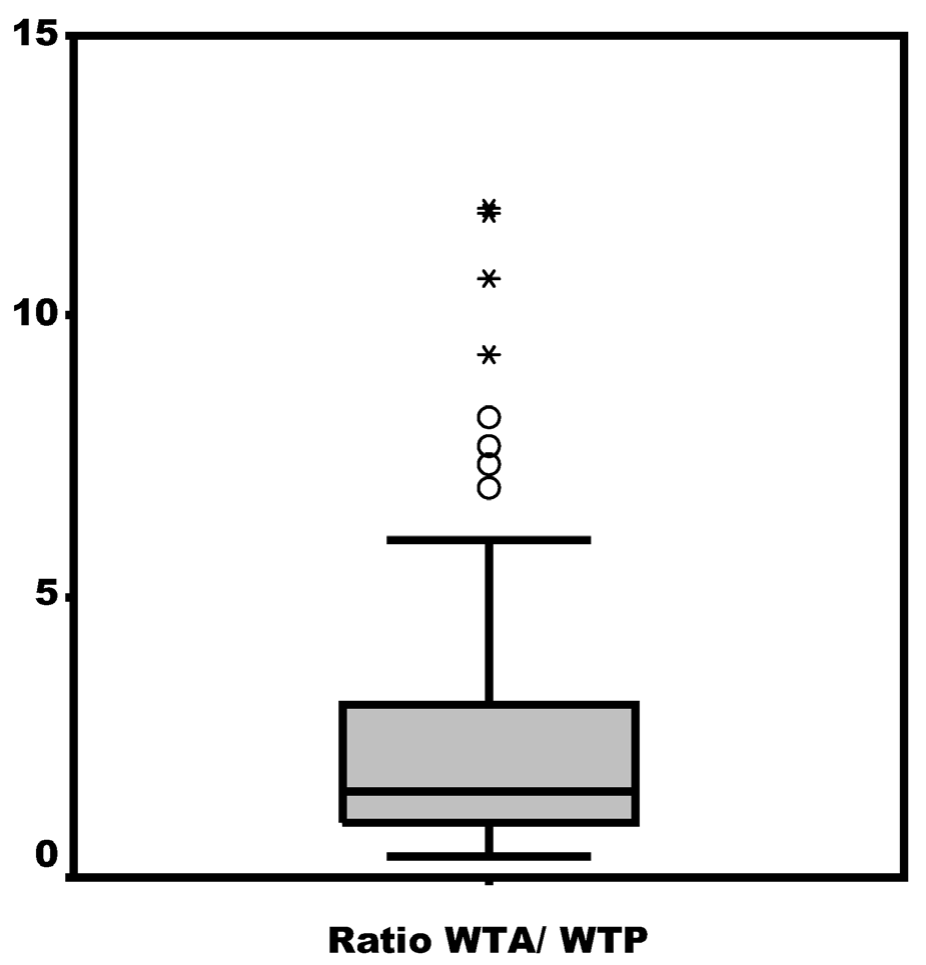
Distribution of the Willingness to Accept (WTA)/Willingness to Pay (WTP) ratio*. *Not shown are 18 values greater than 15.

**Figure 2:**
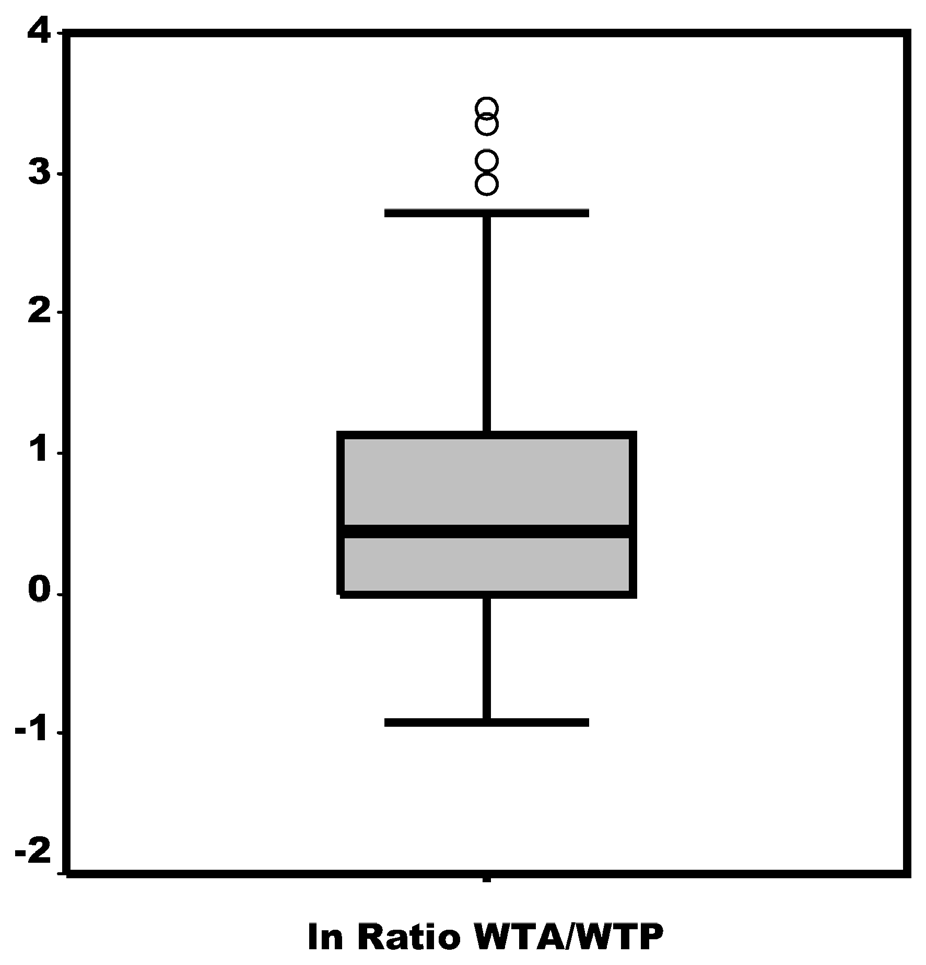
**Distribution of the smoothed WTA/WTP variable (ln [WTA/WTP])**.

The distribution of the WTA/WTP ratio in the 404 subjects who expressed values different from zero is asymmetrical to the right (Figure 1). The mode is worth 1 and includes 36.6% of the responses. Eleven values are less than 1 (2.7%) and 81 (20.0%) are greater than 4. Median distribution is 1.55 with an interquartile range (1.00-3.08). The mean WTA/WTP ratio is 3.30 (CI 95%: 2.84-3.75). Figure 2 shows the distribution of the "smoothed" variable (ln WTA/WTP).

The explicative model is summarized in Table 2. The ln of the WTA/WTP relationship increases with age and in income areas in the lower distribution tercile of the Community of Madrid, decreases in social classes with more specialized activities, as adjusted family income increases, and in persons with chronic illnesses. The model explains 20% of the variability of the ln of the WTA/WTP ratio. In relative terms, social class by itself provides 30% of the model's explicative capacity, age 22%, area income and family income 10% each, and the existence of chronic pathologies less than 3%.

**Table 2:** Summary of the explicative model without including zero WTP or WTA values.

	Unstandardized coefficients		t	**Sig**.	95% LLCI	95% ULCI	VIF
						
	B	Robust **S.E**.					
**Constant**	0.473	0.229	2.06	0.040	0.021	0.924	

Social Class*							

**Group I**	-0.619	0.166	-3.72	<0.001	-0.946	-0.291	1.94

**Group II**	-0.739	0.162	-4.56	<0.001	-1.058	-0.420	1.90

**Group III**	-0.534	0.167	-3.20	0.001	-0.863	-0.206	1.74

**Group IV**	-0.537	0.147	-3.65	<0.001	-0.826	-0.247	2.29

**Age**	0.016	0.003	4.77	<0.001	0.009	0.022	1.41

**Income in the Area (Lower vs Upper tercile)**	0.288	0.092	3.14	0.002	0.108	0.467	1.21

**Adjusted family income (by 1000 €)**	-0.123	0.038	-3.23	0.001	-0.198	-0.048	1.33

**Chronic Illness**	-0.178	0.094	-1.89	0.060	-0.364	0.007	1.38

N 404, F 11.31 p < 0.001, Adjusted R^2 ^0.203, Mean VIF 1.65

Dependent variable ln (WTA/WTP)

Robust S.E.: Robust standard error estimations

Sig: Significance

95% LLCI: Lower Limit of the B 95% Confidence Interval

95% ULCI: Upper Limit of the B 95% Confidence Interval

VIF: Variance Inflation Factor

* Reference for Social Class: Group V

For every ten years of age increase, the WTA/WTP relationship increases a mean of 0.17 points (e^10× 0.016^). Living in areas with lower average disposable incomes increases this relationship a mean of 0.33 points. Having a job in the group I in the classification proposed (managers, directors) with respect to people in group V (unskilled manual workers) represents a mean decrease in the WTA/WTP relationship of 0.46 points (1-e^-0.619^). The WTA/WTP ratio decreases 0.12 points for every additional thousand-euro increase in disposable family income. People with chronic illnesses present a WTA/WTP ratio that is 0.16 points lower, although this relationship is at the edge of signification (p = 0.060).

Other patient characteristics related to perception of health status, accessibility to the service, or satisfaction with it, or with risk perception, were not explicative.

Taking WTA/WTP as a dependent variable, including values estimated for zero responses, does not significantly modify the model. The magnitude of the coefficients of signification did not vary either (Table 3).

**Table 3:** Summary of the explicative model substituting zero responses for WTP or WTA for their estimated values.

	Unstandardized coefficients		t	**Sig**.	95% LLCI	95% ULCI	VIF
						
	B	Robust **S.E**.					
**Constant**	0.447	0.213	2.09	0.037	0.026	0.867	

Social Class*							

**Group I**	-0.581	0.161	-3.61	<0.001	-0.897	-0.265	1.95

**Group II**	-0.653	0.159	-4.11	<0.001	-0.966	-0.340	1.91

**Group III**	-0.455	0.161	-2.81	0.005	-0.773	-0.137	1.78

**Group IV**	-0.424	0.145	-2.92	0.004	-0.709	-0.138	2.33

**Age**	0.015	0.003	5.05	<0.001	0.009	0.021	1.44

**Income in the Area (Lower vs Upper tercile)**	0.242	0.084	2.86	0.004	0.076	0.408	1.20

**Adjusted family income (by 1000 €)**	-0.117	0.035	-3.32	0.001	-0.186	-0.047	1.34

**Chronic Illness**	-0.221	0.087	-2.53	0.012	-0.393	-0.050	1.39

N 451, F 10.72, p < 0.001, Adjusted R^2 ^0.185, Mean VIF 1.67

Dependent variable ln (WTA/WTP)

Robust S.E.: Robust standard error estimations

Sig: Significance

95% LLCI: Lower Limit of the B 95% Confidence Interval

95% ULCI: Upper Limit of the B 95% Confidence Interval

VIF: Variance Inflation Factor

* Reference for Social Class: Group V

If we construct an alternative model with the WTA-WTP difference as dependent variable, obviating the asymmetrical distribution, the explicative variables are the same, although the adjustment of the model is poorer.

## Discussion

The perception of the value of service received in primary care in a public health system differs from the WTP and WTA perspective. There is a debate about what the appropriate measurement is to determine the perception of value of a good or service when the enjoyment of the good or service already exists. The theory suggests that anyone with a right to the status quo should have any damages they suffer, relative to that position, valued using WTA [25]. While this situation is acknowledged, WTA is not widely used to value damages. On the other hand, what constitutes 'rights' is not easy to determine, especially in this case, in which access to the service is provided by "insurance". The NOAA Panel suggests that WTP always be utilized to evaluate a good or service. It is commonly argued that this constitutes the most conservative, and therefore, preferred, option, because, in the most unfavourable case the WTP can mark the lower limit of valuation [26]. We cannot provide an answer to whether WTP or WTA are more realistic approaches for the evaluation of the service received from the primary care physician. What is established is that the WTA/WTP ratio for the visit to the family physician in a public health system varies with the characteristics of certain individuals, tending to be higher in older people, or in less favoured social classes, or in subjects with lower income or living in more depressed areas.

In the scenario presented the median WTA/WTP ratio is situated at 1.55, while the mean is 3.30, in the range of values found in other studies when referring to goods in the field of health [3,5,6,27]. In a classic study, the evaluation of a new drug to treat cancer showed a WTA/WTP relationship of approximately 2 [3]. The valuation of surgical techniques, a cochlear implant, in a population of children, produced values for this relationship approaching 4 [6]. In another paper on the treatment of drug-dependent persons, the WTA/WTP ratio was situated at about 1.3 [27]. When valuing providing care to the chronically ill the relationship between WTA and WTP closely approaches the unit [5]. In a general context, it was already known that differences between WTA and WTP were greater in goods that could not be found in ordinary markets, or in the case of public goods, than when the experiences referred to goods easier to find in the market. This effect also appeared to maintain itself for whatever design employed [8]. Thus the WTA/WTP relationship observed in our case is found in the range obtained for goods or services the suppression of which could entail some resistance from users.

The interpretation of the WTA/WTP relationship must be made in the real context of the service evaluated and in the theoretical framework described. The service is characterized by a high consumption of resources. In the Community of Madrid there are more than thirteen million annual visits in PC corresponding to almost 63% of the population census [28]. Even though this service does not have a direct cost at the time of use, the user has full perception of its value [21], but requires higher economic reimbursement to go without the service than what this person would be willing to pay for it. If we accept that aversion to loss plays a role in the WTA/WTP relationship, we could point out that the users studied are more reticent of the substitution of the service the older they are and when they come from less favourable socioeconomic situations.

The study of the personal characteristics associated with a higher WTA/WTP ratio situates us in the theoretical framework presented. Economic situation has clear implications for the WTA/WTP relationship, independently of the other factors studied. This situation is expressed by social class that includes professional activity, disposable family income as well as place of residence.

Social class with a more specialized professional activity relates to a reduction of the WTA/WTP ratio. In these groups the limitation of WTP by income is lower and we can assume greater information about the good that will substitute the one being evaluated. Favoured socioeconomic groups or with higher educational levels have always been proposed as a variable negatively related to the WTA/WTP ratio.

Subjects with higher income and who live in areas with higher average incomes also express WTP values closer to WTA. Although both variables could be correlated [29], the model does not detect apparent collinearity, and they can offer complementary information. On the one hand, the limitation of resources can widen the WTA-WTP distance [5,11]. This is deduced from the model proposed, which is congruent with the fact that a lower WTP is associated with higher WTA/WTP quotients. However, living in a certain area of residence provides other additional information. Subjects who live in lower-income areas can have less information about the services proposed as an alternative, and an even lower capacity of finding them. In the total sample, almost one in four subjects has another type of insurance, but this circumstance occurs in 36% of the subjects who live in high-income areas and in 11% of those who live in low-income areas. Some studies have shown that it is easier to offer a WTA in those subjects who have private insurance [6]. It has already been described that patients with more experience in the purchase or enjoyment of a good, as may occur with those who have private health care insurance, present a more limited "aversion to loss", than those who lack this experience [30]. In general, the difficulty in finding a substitute good [9,10] the lack of information and cost of obtaining it [11], and uncertainty [13], have been pointed out as factors that may explain the differences between WTA and WTP.

This circumstance can help interpret a result that in hindsight was surprising. Subjects with chronic illnesses express a WTA/WTP ratio approaching the unit. Initially, one might think that persons with more objective "need" would be more resistant to loss. But they also have greater information about the health system's resources, because their state of health obliges them, generally speaking, to receive other types of services in addition to those provided by the family physician. This could contribute to minimizing the sense of loss, as there is the capacity to substitute the good to do without, namely health care.

Age is a variable that independently increases the value of the WTA/WTP ratio. The "aversion to loss" seems to be greater in older persons and in those with low cultural levels in experimental settings [31], which matches with our results.

We have not found a relationship between exposure to risk and the WTA/WTP ratio. It may be that the communication of a risky behaviour is not easily accepted in an interview, as it affects areas of privacy, or that the variables studied are not sufficiently relevant to define the attitude towards risk. But it should also be noted that the aversion to loss can be independent of the amount of risk the situation requires to be assumed [31].

This paper may present certain limitations, such as those related with the scenario chosen, with the format of the question, with the response and with the explicative model. The model was conceived from an ex-post perspective. The ex-ante perspective is theoretically more correct when obtaining WTA or WTP, but we wanted experience of use to be real, and to have as realistic a description of the scenario as possible. An unrealistic scenario could be the cause of weakness in a study such as this one [32]. We consider this scenario to be sufficiently realistic, having been posed after having received the service evaluated. The payment card format causes the user to behave as if he or she would be in a setting in which the same product was being sold at different prices [32]. Its suitability, and the advantages and inconveniences compared with other formats and other methods of estimating the WTP or WTA have been widely discussed [32,33], but it is a commonly accepted tool. However, the questions about WTA and WTP were posed consecutively, which may cause the valuation from one of the two perspectives to have the other as a reference; the WTA/WTP relationship may be biased towards one [31]. To a certain extent this may have occurred in this case because one in three subjects presented a WTA/WTP relationship exactly equal to one. Another aspect that deserves consideration is the expression of zero responses, which may express a possible rejection of the question posed ("zero protest"). It is fair to expect this situation in the context of services without cost at the time of use. Other papers that sought the declared value of certain health services, or their improvement, found an even higher number of zero responses [20,34,35]. The substitution of zero values for others corresponding to persons with the same characteristics does not have repercussions in the final results of the model, which vouches for its solidity.

With respect to the model's explicative capacity, barely 20%, this can be described as limited. It would not be improved by adding new explicative variables to the model, because objective variables cannot fully explain personal choice or the perception of wellbeing, as recognized in other papers [5].

This paper makes a several relevant contributions. On the one hand, we found that the evaluation of public health services made from the perspective of gain or of loss is different, and this should be taken into account if patient preferences are to be incorporated to health planning [14,15]. On the other hand, we can establish a profile of subjects who could show greater resistance to go without the visit to the family physician in a public-health-service setting, and we found that they present characteristics coherent with the proposals in the theoretical framework in which the study is set. This fact should be taken into consideration when planning primary care services, especially if there is an effort to provide an alternative to the service-providing system described in this paper.

## Conclusions

The valuation of the visit to the family physician in our setting is higher from the perspective of willingness to accept compensation than from the perspective of willingness to pay, and this relationship increases in older persons, with less specialized jobs, who live in less favoured areas, or with fewer economic resources. Future studies can establish the influence of the perception of risk in the valuation of the service received, or the relationship of quality perceived with resistance to loss.

## Competing interests

The authors declare that they have no competing interests.

## Authors' information

Ma Isabel del Cura-Gonázlez is Associate Professor at Rey Juan Carlos I University. Tomás Gómez-Gascón is Associate Professor in the Faculty of Medicine, Complutense University of Madrid. Julia Domínguez-Bidagor is the Clinical psychologist responsible for Health Education at Madrid Health Service. Milagros Beamud-Lagos and Francisco Javier Pérez-Rivas are Associate Professors at the University School of Nursing.

## Authors' contributions

JMF: conceived and participated in the design of the study, performed the statistical analysis, was involved in the discussion and the interpretation of the data and draft the manuscript. MICG: participated in the design of the study, reviewed the statistical analysis and the interpretation of the results and the final manuscript. TGG: was involved in the design of the study, and reviewed the interpretation of the results and the final manuscript. JOM: participated in the statistical analysis, the interpretation and discussion of the results and reviewed the final manuscript. JDB: participated in the design of the study, helped to discuss the results and review the final manuscript. MBL: contributed to the design of the study and to the interpretation and discussion of the results and reviewed the final manuscript. FJPR: contributed to the interpretation and discussion of the results and reviewed the final manuscript

## Pre-publication history

The pre-publication history for this paper can be accessed here:

http://www.biomedcentral.com/1471-2458/10/236/prepub

## Supplementary Material

Additional file 1 **Questionnaire**. This is the questionnaire used in the study in a .doc format. Click here for file
