# Supplementary material for: Differences between willingness to pay and willingness to accept for visits by a family physician: A contingent valuation study
Source: BMC Public Health. 2010 May 10;10:236. doi: 10.1186/1471-2458-10-236 (PMC2883536; doi:10.1186/1471-2458-10-236)
Supplement: Additional file 1 — Questionnaire. This is the questionnaire used in the study in a .doc format. [file 1471-2458-10-236-S1.DOC]

**Economic study of the activity of the family physician by means of contingent valuation and analysis of the explicative factors.**

Dear Sir or Madam:

At this time we are asking you to participate in a study that seeks to assign an economic value to the care activity of the family physician in the field of primary care.

Your participation consists in an interview lasting 15-20 minutes, in which hypothetical scenarios will be posed to and you will be asked how much you think certain products are worth.

The situations are theoretical and in no case do they mean that the visits made will cost you anything now or in the future. This is an exercise to enable establishing relations between characteristics of users of the health system and how they value this service. Your answers will enable us to place a perspective on how people view this, which, in turn, can serve to improve decisions when investing resources in health. At present information of this kind is lacking, which makes your participation very important.

On choosing you for this interview your physician will have noted the number of times you have visited in the last year and, generically, the number of chronic illnesses you suffer, if applicable. At the end you will be asked to provide a series of personal characteristics, the type of profession you hold, your job situation and your income. No information will be registered that would enable identifying you (neither name, telephone, nor address). The information will be stored anonymously, that is, once the interview is over, no one will be able to identify who gave the answers. Treatment of the data will be aggregate; only the answers of all the interviewed people are of interest, not each particular case.

If you have any questions now is the time to ask the interviewer.

In any case, we thank you for your time.

Mr./Ms. ________________________________________________________, with National Identity Document ________, states he/she knows that the information he/she is being asked will be utilized in the development of the project “Economic study of the activity of the family physician be means of contingent valuation and analysis of the explicative factors of the willingness to pay,” and hereby states that he/she has been informed and given his/her consent to participate in this study and that this participation solely consists in taking part in an interview.

Signed

At ____________________ on ______ of _______________________ of 200 .

**SECTION I**

Health is a fundamental right of any person, which is guaranteed by the basic norms of our country. Universal health care is one of the pillars of the so-called “welfare state.” The constitution of the health systems that guarantee this health care is a relatively modern attainment in our history. The development and maintenance of the health systems requires the investment of many resources, which, in our country, come all from taxes. The political class, in its act of governing, decides the percentage of investment of these resources in different social needs (health, justice, education, security, etc.) with criteria that are not explicit, and on occasions, variable in time. Even so, the percentage levels of investment in health in our country are similar to those around us.

There are many factors that influence decisions about the amount of investment in health in a country, but a fundamental one is the perception people have of the need for investment and the results perceived. A special way of valuing the health care process is by attributing an economic value. This attribution can guide, moreover, the decider about the need for investment in each structure of the health system. This idea has led a group of professionals, who work in the public health system, and independently of any interest of the decision-making bodies, to conduct the following study. With it we would like to learn the economic value that primary care users attribute to their visit to the family physician.

The objective of the questions asked below is to learn the perceptions people have and for this they are presented with non-real, imaginary scenarios. In no case should it be assumed that any price is being placed on the service they are being asked about, nothing further from the intent of the researchers.

**Scenario 1.**

Imagine for a moment that there is no public health care system. Suppose you have a health problem similar to what brought you here today to the visit and you have to be attended by the physician who received you today, but you must pay for this care directly.

How much money would you be willing to pay for this visit?

Card 1

** ** A: <20 euros **  B**: > 20 & <40 euros **C**: > 40 euros

Card 2

** ** A: 0 euros

- B: 1- 5 euros
- C: 6- 10 euros
- D: 11- 15 euros
- E: 16- 20 euros
- F: 21- 25 euros
- G: 26-30 euros
- H: 31-40 euros
- I: 41- 50 euros
- J: 51- 60 euros
- K: 61- 70 euros
- L: 71-80 euros
- M: 81-90 euros
- N: More than 90 euros

**Scenario 2.**

If you could not be visited as today for your family physician, how much money do you think you should receive for compensating you? (this is the money to renounce to the service, perhaps you could receive the same attention, using this money)

Card 1

** ** A: <20 euros **  B**: > 20 & <40 euros **C**: > 40 euros

Card 2

** ** A: 0 euros

- B: 1- 5 euros
- C: 6- 10 euros
- D: 11- 15 euros
- E: 16- 20 euros
- F: 21- 25 euros
- G: 26-30 euros
- H: 31-40 euros
- I: 41- 50 euros
- J: 51- 60 euros
- K: 61- 70 euros
- L: 71-80 euros
- M: 81-90 euros
- N: More than 90 euros

**Section II**

**We are going to ask you for information about your state of health and the visit you have just made**

**Your age is ___years**

** Man  Woman**

**You are from …?**

**** Spain

- Rest of European Union plus Iceland, Norway and Switzerland.
- Latin America

****  North Africa

- Sub-Saharan Africa
- Other non-EU European countries

Includes members with deferred incorporation (Bulgaria and Rumania), candidates (Turkey, Croatia and Macedonia) and Albania, Armenia, Azerbaijan, Byelorussia, Bosnia, Georgia, Liechtenstein, Moldavia, Montenegro, Russia, Serbia and Ukraine.

- Asia
- Other:…

**In the last year, have you been admitted to hospital?** (including remaining more than 24 hours in the emergency ward)

**** Yes **** No

**Number of visits to the family physician in the last year**: __times.

**The patient has a chronic pathology :**

**** Yes **** No

**Total number of chronic pathologies:** ___

**If you do not wish to participate in this study, the reason is:**

**** Lack of time

- Not interested in study question
- Prefers not to answer

**** Other (specify)

**How long did you have to wait to obtain an appointment for the visit you have just made?**

**** Same day

- One day
- Two days
- Three days

**** More than three days

**How long did you been wait since your time of appointment to be attended?**

**** Less than 15 minutes

- Between 15 -30 minutes
- Between 31-60 minutes

**** More than 1 hour

**Do you have another system of insurance?**

**** Yes **** No

**Which one?**

**** Asisa

- Sanitas

**** Adeslas

- Other (specify):

**If you do have another system of insurance, who pays for it?**

**** Your company **** Yourself

**As far your state of health today, indicate the answer in each section that best describes it:**

Mobility

**** I have no problems walking

**** I have some problems walking

**** I have to stay in bed

Personal care

**** I have no problems with personal care

**** I have some problems to wash and dress myself

**** I cannot wash and dress myself

Daily activities (e. g., work, study, housework, family activities or during leisure time)

**** I have no problems doing my daily activities

**** I have some problems doing my daily activities

**** I cannot perform my daily activities

Pain/discomfort

**** I have no pain or discomfort

- I have moderate pain or discomfort
- I have much pain or discomfort

Anxiety/depression

**** I am not anxious or depressed

**** I am moderately anxious or depressed

**** I am very anxious or depressed

Compared with my general state of health during last 12 months, my state of health today is:

**** Better

**** Same

**** Worse

To help people describe how good or bad their state of health is we have drawn a scale similar to a thermometer on which 100 marks the best state of health imagined and 0 the worst state of health imagined. We would like you to indicate on this scale, in your opinion, how good or bad your state of health is *today*.

Better

State

of Health

Worst

State

of Health

Your health status today

**As far as relation with your physician, you are given nine statements of what a person can think of this. Pick the one most suitable to each statement with respect to your case marking one number per statement (remember that what you say is confidential and that no one will have access to your answers).**

**1. My physician helps me.**

| 1  Not at all appropriate | 2  Somewhat appropriate | 3  Appropriate | 4  Mostly appropriate | 5  Totally appropriate |
| --- | --- | --- | --- | --- |

**2. My physician has enough time for me.**

| 1  Not at all appropriate | 2  Somewhat appropriate | 3  Appropriate | 4  Mostly appropriate | 5  Totally appropriate |
| --- | --- | --- | --- | --- |

3. I trust my physician.

| 1  Not at all appropriate | 2  Somewhat appropriate | 3  Appropriate | 4  Mostly appropriate | 5  Totally appropriate |
| --- | --- | --- | --- | --- |

4. My physician understands me.

| 1  Not at all appropriate | 2  Somewhat appropriate | 3  Appropriate | 4  Mostly appropriate | 5  Totally appropriate |
| --- | --- | --- | --- | --- |

5. My physician is dedicated to help me.

| 1  Not at all appropriate | 2  Somewhat appropriate | 3  Appropriate | 4  Mostly appropriate | 5  Totally appropriate |
| --- | --- | --- | --- | --- |

6. My physician and I agree on the nature of my medical symptoms.

| 1  Not at all appropriate | 2  Somewhat appropriate | 3  Appropriate | 4  Mostly appropriate | 5  Totally appropriate |
| --- | --- | --- | --- | --- |

7. I can talk with my physician.

| 1  Not at all appropriate | 2  Somewhat appropriate | 3  Appropriate | 4  Mostly appropriate | 5  Totally appropriate |
| --- | --- | --- | --- | --- |

8. I feel content with my physician’s treatment.

| 1  Not at all appropriate | 2  Somewhat appropriate | 3  Appropriate | 4  Mostly appropriate | 5  Totally appropriate |
| --- | --- | --- | --- | --- |

9. I find my physician easily accessible.

| 1  Not at all appropriate | 2  Somewhat appropriate | 3  Appropriate | 4  Mostly appropriate | 5  Totally appropriate |
| --- | --- | --- | --- | --- |

**Next, we are going to ask you series of questions about activities in daily life. There is no right or wrong answer, and we would only like to know your attitude towards certain situations.**

**In the last month, have you smoked at least one cigarette a day?**

**** Yes **** No

**In the last month, have you ever driven a motor vehicle without the obligatory safety measures (helmet, seat belt, etc.)?**

**** Yes **** No

**In the last month, at any time have you carried out your job without observing the obligatory protection measures (helmet, gloves, protective clothing, safety harness/, etc.)?**

**** Yes **** No

**How often do you drink alcohol?**

**** Never 0 points

**** Once or twice a month 1 points

**** From 2 to 4 times a month 2 points

**** From 2 to 3 times a week 3 points

**** 4 or more times a week 4 points

**How many drinks of alcohol do you normally take in a day?**

**** 1-2 0 points

**** 3-4 1 points

**** 5-6 2 points

**** 7-9 3 points

**** 10 or more 4 points

**How often to you have six or more drinks of alcohol in a single day?**

**** Never 0 points

**** Less than once a month 1 points

**** Monthly 2 points

**** Weekly 3 points

**** Daily or almost daily 4 points

**(add up points of the three questions that refer to alcohol) points**

**4 or more points catalogues the subject as a drinker at risk (click on yes when filling out questionnaire)**

**In the last month, have you consumed any type of controlled substance or used tranquilizing or sleep medication without prescription?**

**** Yes **** No

**In the last month, have you had sexual relations with a person who is not your regular partner without using a condom?**

**** Yes **** No

**Section III**

**Finally, we are going to ask you for some statistical information that will help us classify your answers and interpret the results of the study**

**The number of persons who live in my home is __ persons**

**Your main occupation at present is**

**** Housewife.

**** Student.

**** Worker.

**** Unemployed.

- Retired.

**The highest level of studies you have completed is**

**** I don’t know how to read or write.

**** No education.

**** Primary studies.

- Secondary studies
- Superior studies

**My last paying job was:**

**** I Manager, director, liberal profession.

**** II Mid-level positions and sales managers.

**** III Non-manual skilled worker.

**** IVa Skilled manual worker.

**** IVb Partially-skilled manual worker.

**** V Non-skilled manual worker.

In case of not having a paid job the highest class within the family unit is considered. The unemployed or temporary or permanent incapacity or retired are classified according to the last job held.

**The monthly income of your family unit (adding up all family members who live in your home) is between the range indicated with the letter:**

**** A: Less than 600 euros

- B: 600- 1200 euros
- C: 1200-1800 euros
- D: 1800-2400 euros
- E: 2400- 3600 euros
- F: 3600- 4800 euros
- G. 4800-6000 euros
- H: 6000-7200 euros
- I: More than 7200 euros

Note: Income per person is calculated by the formula $R=\frac{Rt}{N^{0.4}}$

Rt. Gross family income. N number of family members

The exponent 0.4 is a correction factor that reflects economy of scale.
